# Primary Sternal Osteomyelitis with Acute Mediastinitis, Successfully Treated with Minimally Invasive Surgical Drainage

**DOI:** 10.70352/scrj.cr.25-0056

**Published:** 2025-07-19

**Authors:** Iori Tsuji, Fumihiko Kinoshita, Yoshiyuki Nakanishi, Takaki Akamine, Mikihiro Kohno, Keigo Ozono, Tomoyoshi Takenaka, Tomoharu Yoshizumi

**Affiliations:** 1 Department of Surgery and Science, Graduate School of Medical Sciences, Kyushu University, Fukuoka, Fukuoka, Japan; 2 Department of Thoracic Surgery, Kyushu University Hospital, Fukuoka, Fukuoka, Japan

**Keywords:** primary sternal osteomyelitis, acute mediastinitis, mediastinal abscess, surgery

## Abstract

**INTRODUCTION:**

Primary sternal osteomyelitis (PSO) is a rare disease that occurs without any contiguous focus of infection, and there are few reports of acute mediastinitis due to PSO. In this report, we describe a case of PSO with acute mediastinitis successfully treated with a minimally invasive approach.

**CASE PRESENTATION:**

A 71-year-old man visited his local doctor for anterior chest pain. He had no history of trauma or chest surgery. He was treated conservatively because of a few abnormalities on CT. However, his symptoms worsened, and a CT was re-taken 13 days later. The CT showed an abscess on the left side of the anterior mediastinum and subcutaneous tissues, as well as destruction of the sternum. With the diagnosis of acute mediastinitis and mediastinal abscess, thoracoscopic and subcutaneous drainages of the abscess were performed. After surgery, blood culture examination showed methicillin-sensitive *Staphylococcus aureus*, and we judged the mediastinitis to be caused by PSO. The thoracic drain was removed on postoperative day 39, the open subcutaneous wound closed spontaneously, and the patient was discharged on postoperative day 45. He continued oral antibiotics for the next 2 months, and the abscess cavity completely resolved.

**CONCLUSIONS:**

Although PSO is rare, it can lead to mediastinitis and should be suspected when anterior chest pain is present. Our case of PSO with acute mediastinitis progressed rapidly over a few days but could be treated with minimally invasive thoracoscopic and subcutaneous drainages, without the need for invasive sternal debridement and drainage.

## Abbreviations

CRP
: C-reactive protein
DSWI
: deep sternal wound infection
HbA1c
: hemoglobin A1c
IV
: intravenous
MSSA
: methicillin-sensitive *Staphylococcus aureus*
NPWT
: negative pressure wound therapy
PSO
: primary sternal osteomyelitis
WBC
: white blood cell

## INTRODUCTION

Acute mediastinitis is a relatively rare but fatal disease, characterized by an inflammatory process in the mediastinum. There are multiple causes of acute mediastinitis. Acute mediastinitis after midline sternotomy is the most common cause.^1,2)^ In contrast, nonsurgical acute mediastinitis is most often caused by esophageal rupture or by descending necrotizing mediastinitis resulting from the spread of infection from the head and neck region.^2,3)^

Acute mediastinitis is associated with a high morbidity and mortality. Although the mortality rate of acute mediastinitis has improved from a reported 49% in 1938 to 10%–20% in recent years, it has remained high.^3–5)^ In a recent report, a joint study by the Japan Broncho-Esophagological Society and the Japanese Association for Chest Surgery (JBES1703/JACS1806 study) showed that 8 of 225 patients (3.6%) with acute mediastinitis died within 30 days, and the 5-year overall survival rate was 68.6%.^6)^ Therefore, it is necessary to improve the outcomes of acute mediastinitis.

While secondary sternal osteomyelitis is a relatively common condition following sternotomy, PSO is an uncommon disease.^7–9)^ The incidence of PSO is reported to be about 0.3% of all osteomyelitis cases.^7–9)^ Among the cases of PSO, few cases of mediastinitis have been reported, and the treatment strategy for PSO has not yet been established. In this report, we describe a rare case of PSO with acute mediastinitis that occurred very rapidly and was successfully treated with minimally invasive surgical drainage.

## CASE PRESENTATION

**Fig. 1:**
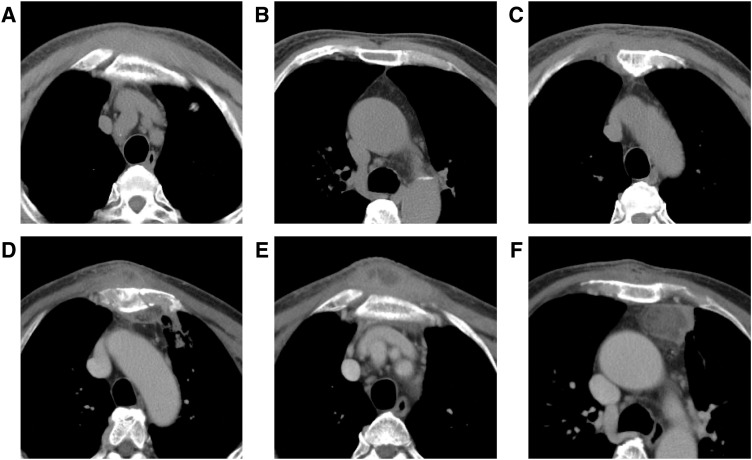
The first CT scan, which was performed at a local clinic, showed no specific findings in the subcutaneous tissue of the anterior chest (**A**), anterior mediastinum (**B**), or sternum (**C**). The 2nd CT scan, which was performed 13 days after the previous CT scan, showed destruction of the sternum (**D**) with the abscesses in the subcutaneous tissue of the anterior chest (**E**) and on the left side of the anterior mediastinum (**F**).

A 71-year-old Japanese man visited his local doctor for anterior chest pain that had lasted for 2 weeks. He had no history of trauma or surgery, including to the chest. A CT scan showed no specific findings (**Fig. 1A**–**1C**), and he was prescribed celecoxib and treated conservatively. However, 9 days after the conservative treatment, his symptoms worsened, and he visited an orthopedic surgeon at our hospital. Physical examination revealed swelling and tenderness on the central anterior chest in front of the sternum. Laboratory data revealed that WBCs and CRP were elevated (WBCs = 9.42 × 10^3^/mm^3^; CRP = 16.41 mg/dL), and serum glucose and HbA1c were also high (serum glucose = 308 mg/dL; HbA1c = 8.1%). A 2nd CT scan, which was performed 13 days after the previous CT scan, showed destruction of the sternum (**Fig. 1D**) and abscesses in the subcutaneous tissue of the anterior chest (**Fig. 1E**), as well as on the left side of the anterior mediastinum (**Fig. 1F**). He was referred to our department with a diagnosis of acute mediastinitis and mediastinal abscesses. We decided to perform drainage of the mediastinal abscesses by both surface and thoracoscopic approaches, instead of incising and draining the sternum from the surface, to minimize the invasiveness of the procedure.

**Fig. 2:**
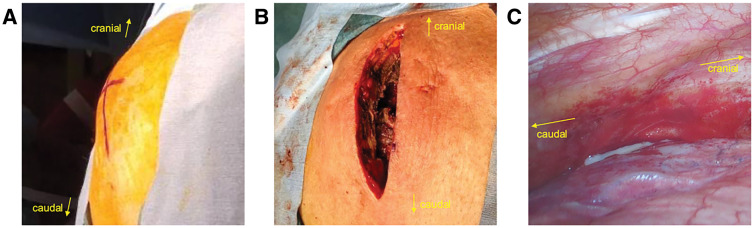
During the surgery, the swollen anterior chest (**A**) was opened, and drainage of the abscess in the subcutaneous tissue of the anterior chest was performed (**B**). Then, under thoracoscopy, adhesions between the left upper lobe and chest wall were observed (**C**). The adhesions were dissected and the mediastinal pleura was incised to open and drain the mediastinal abscess.

First, the patient was placed in the supine position, and the anterior chest abscess cavity was opened to drain the abscess (**Fig. 2A** and **2B**). The wound of the anterior chest was left open and packed. Then, the patient was repositioned to the right lateral position, and the anterior mediastinal abscess was opened and drained under thoracoscopy (**Fig. 2C**). There were adhesions between the left upper lobe and the chest wall. We bluntly dissected the adhesions and incised the mediastinal pleura, and the mediastinal abscess cavity was opened and drained. Two 24-Fr thoracic drainage tubes were placed in the abscess cavity and dorsally in the left thoracic cavity, respectively.

**Fig. 3:**
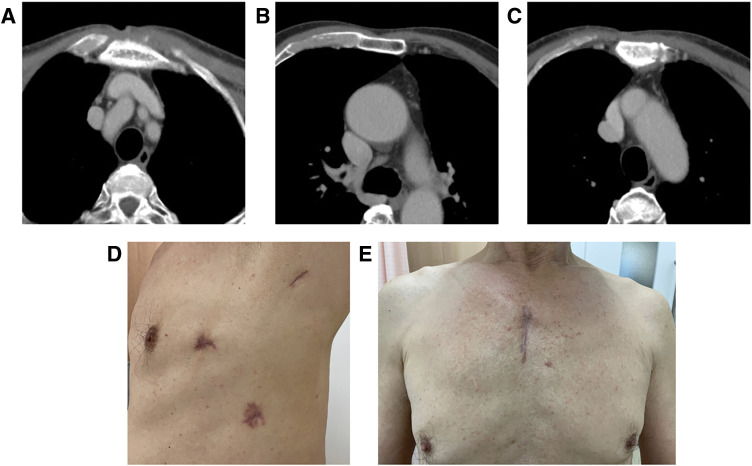
The CT scan, which was taken on postoperative day 135, showed improvement of the abscesses in the subcutaneous tissue of the anterior chest (**A**) and the anterior mediastinum (**B**). The destruction of the sternum was also improved (**C**). The surgical wounds on the lateral chest (**D**) and the anterior chest (**E**) are shown.

On the day of surgery, both bacterial cultures of the anterior mediastinal abscess and blood, which were collected on the same day, were positive for *Staphylococcus aureus*, and we judged the mediastinitis to be caused by PSO. Since no anaerobic bacteria were detected, we concluded that the possibility of a descending origin was low. Vancomycin (0.75 g IV every 12 hours) and daptomycin (350 mg IV every 24 hours) were administered in consideration of methicillin-resistant *Staphylococcus* *aureus*. Continuous washing of the abscess cavity through the drainage tube was started, and glycemic control was initiated using a combination of rapid-acting and long-acting insulin on the day after surgery. On POD 2, susceptibility testing revealed that the causative organism was MSSA, and the antibiotic was changed to cefepime (2 g IV every 8 hours). On POD 4, an echocardiogram was performed to rule out infective endocarditis and revealed no obvious vegetations. Metformin was initiated at a dose of 500 mg on POD 7, and insulin was discontinued on the same day because of satisfactory glycemic control. A dipeptidyl peptidase-4 inhibitor was added at a dose of 50 mg on POD 9. Subsequently, on POD 14, the antibiotic was changed to cefazolin (2 g IV every 8 hours), and a CT scan was taken. The abscess cavity had shrunk but remained. Furthermore, the bacterial culture of the abscess cavity fluid also remained positive. Then, CT-guided drainage was performed and an additional drain was placed in the anterior mediastinal abscess on POD 22, and levofloxacin (500 mg IV every 24 hours), which is highly translocatable to bone marrow, was additionally administered. By this time, WBC and CRP had almost normalized (WBCs = 3.99 × 10^3^/mm^3^; CRP = 1.20 mg/dL). The left dorsal thoracic drain was removed and the dose of metformin was reduced to 250 mg on POD 29, and a CT scan was re-taken on POD 31. The abscess cavity was found to be reduced, and the anterior mediastinal drain inserted under CT guidance was removed on POD 32. Furthermore, metformin was discontinued because of good glycemic control. The left ventral thoracic drainage tube was also removed on POD 39. Cefazolin was discontinued, and levofloxacin was switched from intravenous to oral administration (500 mg per os every 24 hours), and the patient was discharged home on POD 45. After discharge, the patient continued to take levofloxacin, and a CT scan was performed on POD 135. The abscesses in the subcutaneous tissue of the anterior chest and the anterior mediastinum had disappeared (**Fig. 3A** and **3B**), and the destruction of the sternum had improved (**Fig. 3C**). The surgical wound is shown in **Fig. 3D** and **3E**. The patient’s symptoms also disappeared, and the administration of antibiotics was completed.

## DISCUSSION

In this case report, a patient with no history of trauma or chest surgery developed a rapidly progressive acute mediastinitis and a mediastinal abscess with destruction of the sternum. Although drainage of the abscess by splitting the sternum was considered, drainage from the surface of the anterior chest and the left thoracic cavity could provide a minimally invasive treatment with good therapeutic efficacy.

The cause of acute mediastinitis and mediastinal abscess in this case was considered to be PSO. PSO is sternal osteomyelitis with no identifiable adjacent infection sites, such as thoracic surgery, chest trauma, or subclavian vein catheterization.^7–10)^ Several risk factors for PSO, such as human immunodeficiency virus infection, obesity, diabetes mellitus, and substance abuse, have been reported.^11,12)^ In our case, admission blood tests revealed untreated diabetes mellitus, which seemed to be a risk factor for PSO, and no other risk factors were identified.

The symptoms of PSO include anterior chest pain, swelling, erythema, and persistent fever. However, these symptoms are nonspecific and do not always appear, making the diagnosis of PSO difficult.^7–13)^ In this case, the patient presented with anterior chest pain and swelling; however, the initial CT showed minimal changes, and conservative treatment was performed without a definitive PSO diagnosis. Subsequent CT imaging only 13 days later revealed rapid sternal destruction with mediastinal abscess. Therefore, if conservative treatment had been continued without follow-up CT, the outcome could have been fatal. When the response to conservative treatment is poor and anterior chest pain is progressive, though rare, PSO needs to be taken into consideration.

There have been few reports of mediastinitis associated with PSO.^14,15)^ While there is no established treatment for mediastinitis associated with PSO, drainage of the abscess is generally considered essential.^14,15)^ Multiple reports exist on sternal osteomyelitis with mediastinitis as a complication of median sternotomy.^2)^ The treatment strategy for DSWI involving destruction of the sternum after median sternotomy for cardiac surgery is sternal debridement and reconstruction with muscle flaps.^2)^ Although this case was PSO and not a postoperative complication, when considering a treatment strategy similar to that for DSWI, invasive treatment such as sternal debridement, drainage, and chest wall reconstruction with muscle flaps seemed to be necessary. In cases of primary osteomyelitis other than of the sternum, hematogenous infection is typically the underlying cause, and antibiotic therapy serves as the mainstay of treatment. However, in the presence of abscess formation or bone destruction, surgical interventions such as drainage or debridement may be required.^16,17)^ In this case, significant treatment effectiveness was achieved through less invasive approaches. Including the present case, there have been 3 reported adult cases in which PSO progressed to mediastinitis, which are summarized in **Table 1**. The thoracic approach for drainage in mediastinitis due to PSO may be unique to this case. This method offers the advantage of accessing the abscess without sternal debridement, and post-treatment CT scans showed improvement in sternal destruction, suggesting the efficacy of this minimally invasive approach. The thoracic approach for mediastinitis due to PSO may be an effective treatment option.

**Table 1:** Including the present case, there have been 3 reported adult cases in which primary sternal osteomyelitis progressed to mediastinitis

Case report	Age/sex	Risk factors	Causative organism	Treatment	Sternal debridement	Outcome
Baraboutis et al.^14)^ (2008)	51/F	None	*Nocardia nova*	Antibiotics Open biopsy and debridement	Performed	Complete remission
Al Ani et al.^15)^ (2023)	51/M	None	MSSA (*Staphylococcus aureus*)	Antibiotics Two debridements NPWT	Performed	Discharged with wound healing in progress; lost to follow-up
Present case (2025)	71/M	Diabetes mellitus	MSSA (*Staphylococcus aureus*)	Antibiotics Thoracoscopic and subcutaneous drainage	Not performed	Complete remission

F, female; M, male; MSSA, methicillin-sensitive *Staphylococcus aureus*; NPWT, negative pressure wound therapy

While *Staphylococcus aureus* is the most common causative organism in sternal osteomyelitis, cases involving *Pseudomonas aeruginosa*, *Salmonella*, *Klebsiella*, *Candida albicans*, and *Mycobacterium tuberculosis* have also been reported.^7–15,18)^ Identification of the causative organism is important for appropriate antibiotic use. In this case, *Staphylococcus* *aureus* was isolated from both abscess and blood cultures, and was later identified as MSSA. There is no consensus on the duration of antibiotic therapy, but reports of acute PSO treatment often follow protocols for other acute osteomyelitis, with 4–6 weeks of administration.^10,12,13)^ In this case, due to persistently positive mediastinal abscess cultures, the total duration of antibiotic therapy was extended. Intravenous administration was discontinued after 6 weeks, with subsequent oral antibiotic management, and no recurrences were observed.

## CONCLUSIONS

Although PSO is a rare disease, it can rapidly progress and lead to fatal acute mediastinitis. Therefore, PSO should be suspected, particularly in patients with risk factors such as diabetes, when anterior chest pain is progressive despite conservative treatment. Our case of PSO with acute mediastinitis progressed rapidly over a few days but could be treated with minimally invasive thoracoscopic and subcutaneous drainages, without the need for invasive sternal debridement and drainage.

## DECLARATIONS

### Funding

Not applicable.

### Authors’ contributions

IT collected the associated data and edited the manuscript.

FK, YN, TA, MK, and KO participated in the treatment.

TT and TY supervised the writing of the manuscript.

All the authors have read and approved the final manuscript.

### Availability of data and materials

Not applicable.

### Ethics approval and consent to participate

This work does not require ethical considerations or approval. Informed consent to participate in this study was obtained from the patient.

### Consent for publication

Oral informed consent was obtained from the patient for the publication of this case report and accompanying images.

### Competing interests

The authors declare that they have no competing interests.
